# Intracranial meningeal melanocytoma diagnosed using an interdisciplinary approach: a case report and review of the literature

**DOI:** 10.1186/s13256-018-1725-9

**Published:** 2018-06-26

**Authors:** Shoko Gamoh, Takaya Tsuno, Hironori Akiyama, Shinya Kotaki, Tamaki Nakanishi, Kaname Tsuji, Hiroaki Yoshida, Kimishige Shimizutani

**Affiliations:** 1 Health Promotion Division, Public Health Bureau, Osaka City Government, Osaka, Japan; 2 Department of Neurosurgery, Kochi Health Sciences Center, Kochi, Japan; 3 Department of Oral Radiology, Osaka Dental University, Osaka, Japan; 4 First Department of Oral and Maxillofacial Surgery, Osaka Dental University, Osaka, Japan

**Keywords:** Melanocytoma, Computed tomography, Magnetic resonance imaging, Multidisciplinary approach

## Abstract

**Background:**

Meningeal melanocytoma is a rare pigmented tumor arising from leptomeningeal melanocytes. Patients with this tumor might initially consult a dentist because a mass lesion in Meckel’s cave could manifest as dental pain and malocclusion, thereby mimicking temporomandibular disorder. The diagnostic approach, especially using imaging modalities, would be challenging in such cases unless an interdisciplinary approach is used.

**Case presentation:**

Here, we report a case of a 39-year-old Japanese man who had a history of pain and numbness on the left side of his face and malocclusion for 3 months before the initial visit. The diagnosis was primary intracranial meningeal melanocytoma arising from Meckel’s cave.

**Conclusions:**

The process by which the final diagnosis of meningeal melanocytoma was reached highlights the importance of collaboration between the medical and dental disciplines. This case also demonstrates that meningeal melanocytoma has a specific signal pattern on magnetic resonance imaging, including high signal intensity on T1-weighted images and low signal intensity on T2-weighted images.

## Background

Meningeal melanocytoma is a benign pigmented tumor of the central nervous system [1]. This tumor is so rare that its incidence has yet to be reported, and there are few reports on such tumors in the English language literature [2]. The posterior fossa is the most common site involved. To date, there are only three reports of this tumor arising in Meckel’s cave and detailed magnetic resonance imaging (MRI) descriptions are available for only four patients. Here we report the MRI findings in a patient with primary intracranial meningeal melanocytoma (IMM) arising from Meckel’s cave.

## Case presentation

A 39-year-old Japanese man presented with a 3-month history of numbness on the left side of his face. His symptoms had gradually progressed and had become painful in the month before the initial visit. He also complained that sometimes he could not chew on the left side. An examination revealed decreased sensation over the distribution of the left trigeminal nerve that did not respond to nonsteroidal anti-inflammatory drugs or muscle relaxants and was only slightly responsive to carbamazepine. His symptoms were associated with dyskinesia of the left masticatory muscles but there was no clicking sound. His facial expression was symmetrical at rest.

**Fig. 1:**
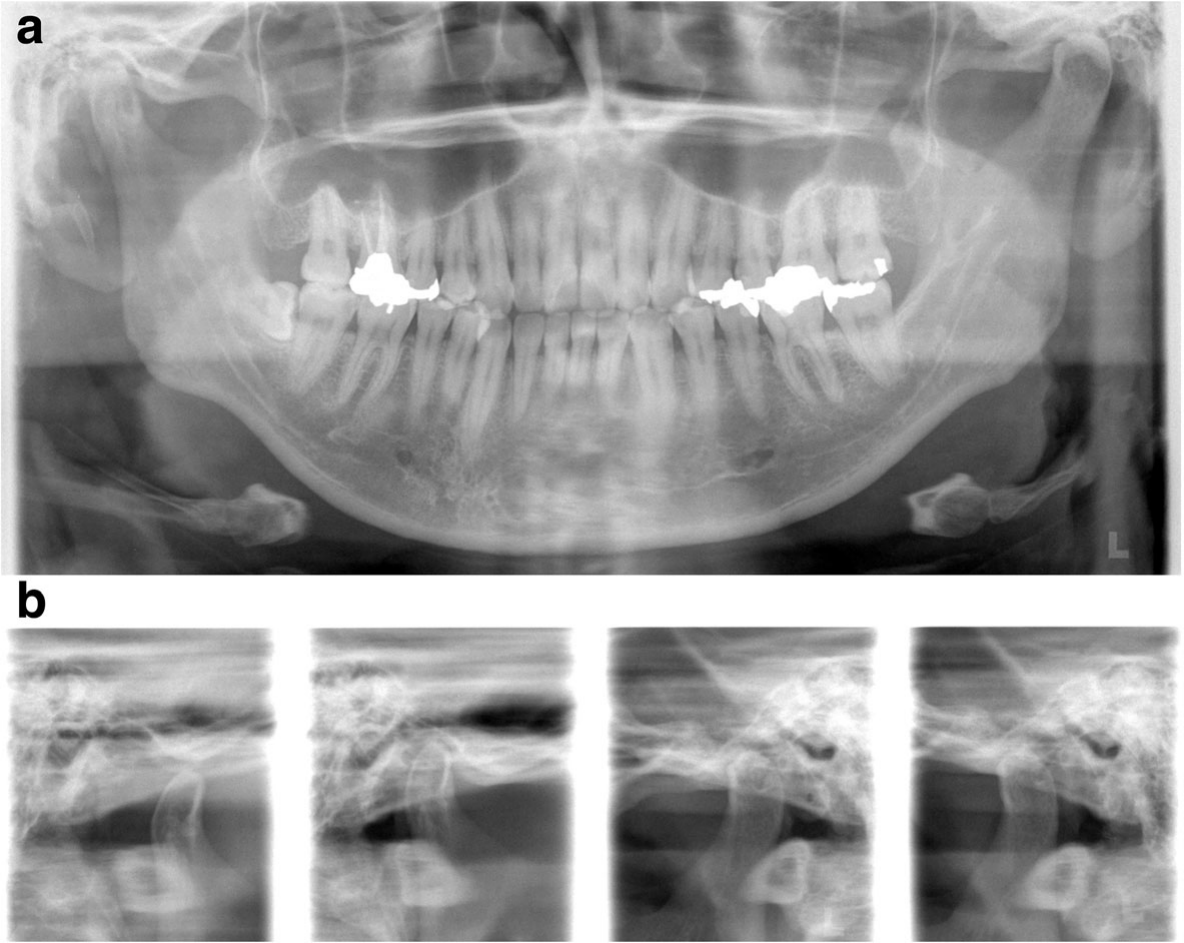
Conventional radiographs. **a** Panoramic radiography showing no particular findings relevant to the symptoms. **b** Panoramic temporomandibular joint projection method demonstrating slight restriction of jaw movement on the left

**Fig. 2:**
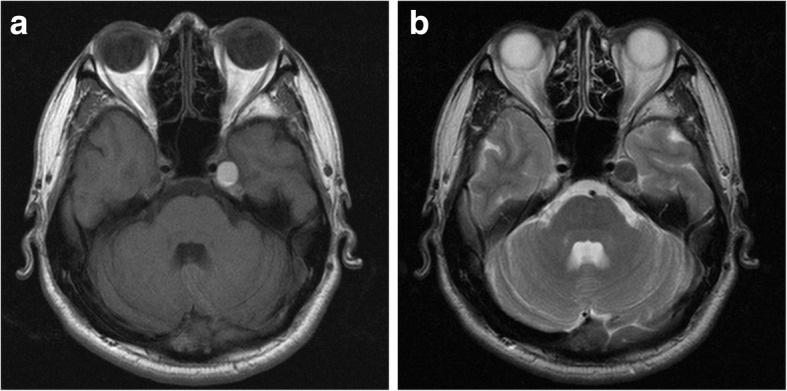
Magnetic resonance imaging demonstrating a tumor 10 mm in diameter. **a** T1-weighted axial images (repetition time 667/echo time 9) revealed a homogeneously high signal tumor in Meckel’s cave. **b** T2-weighted axial images (repetition time 5200/echo time 98) revealed a low signal tumor exerting pressure on trigeminal nerve

**Fig. 3:**
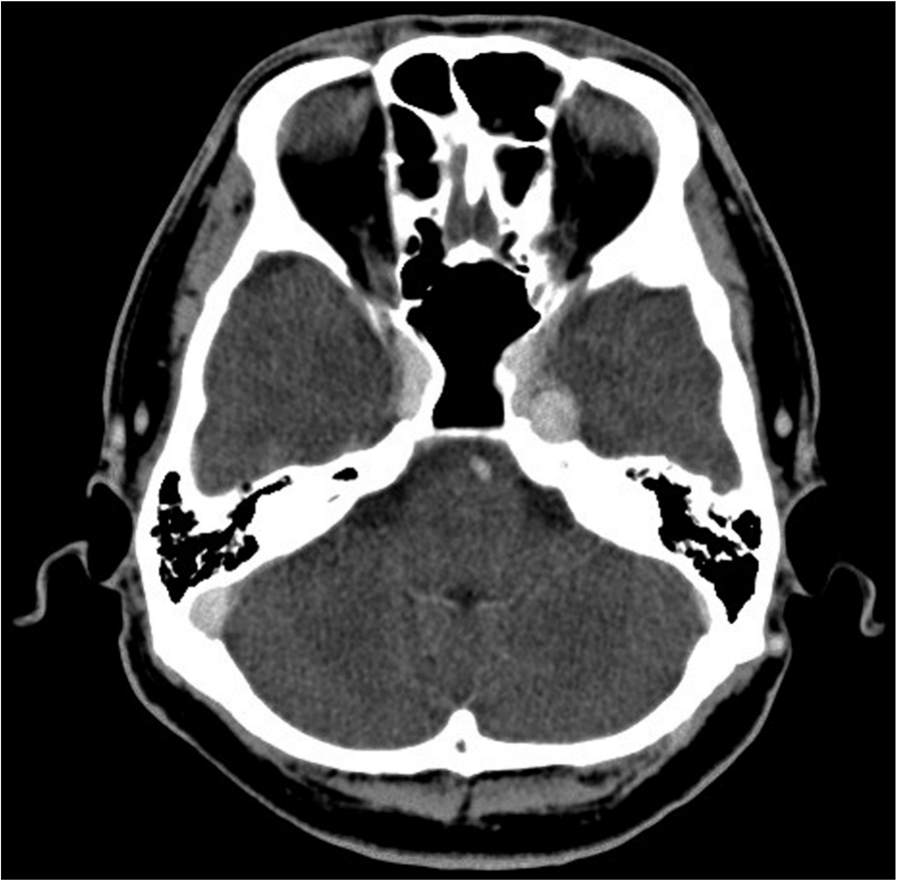
Unenhanced computed tomography image at referral hospital. A localized well-defined mass in Meckel’s cave, homogeneously hyperdense to the gray matter

His past medical history was significant for acute gastritis, duodenal ulcer, and depression, for which brotizolam, flunitrazepam, and paroxetine had been prescribed, respectively. He was reticent and had difficulty communicating his feelings and wishes, which appeared to be related to his history of depression. Panoramic radiography revealed no specific findings relevant to his symptoms (Fig. 1a) but did identify slight restriction of movement of the temporomandibular joint on the left (Fig. 1b). MRI of the temporomandibular joint region was inconclusive for temporomandibular disorder and his symptoms were nonspecific for trigeminal neuralgia. Therefore, we extended the scanning range into the brain region and found a tumor measuring 10 mm in diameter and a homogeneously high signal intensity on axial T1-weighted images compared with gray matter (Fig. 2a) and low signal on axial T2-weighted images (Fig. 2b) in Meckel’s cave. The tumor appeared to be exerting pressure on his trigeminal nerve. He was referred to the neurosurgery department where unenhanced computed tomography (CT) images demonstrated a localized well-defined mass lesion in Meckel’s cave, which was homogeneously hyperdense compared with gray matter. No calcification was present (Fig. 3).

**Fig. 4:**
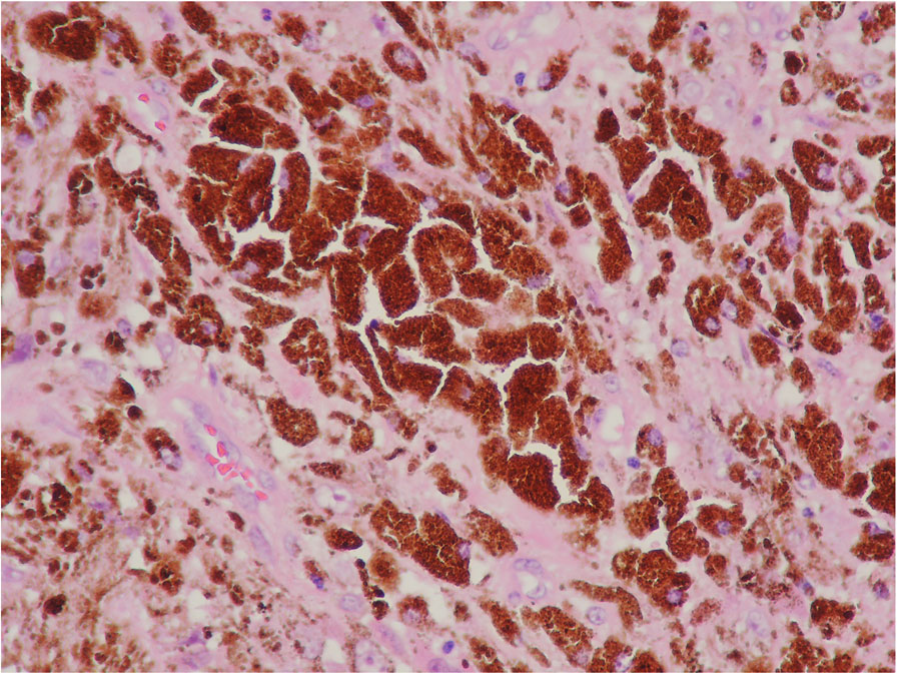
Microscopic photograph with positive staining of the melanocytic marker, human melanoma black-45 (hematoxylin and eosin × 400)

En bloc excision was subsequently performed. Immunohistochemistry was positive for melanocytic features of Melan A (MART1; melanoma antigen recognized by T cells-1), human melanoma black-45, vimentin, and S-100 protein and negative for cytokeratin AE1/AE3 and glia fibrillary acidic protein (Fig. 4). Cellular proliferation was assessed by staining for Ki-67, which was positive, but the index was as low as 1–5%. These findings were associated with proliferation of tumor cells that contained abundant melanin pigment. Based on the above pathology results, a definitive diagnosis of melanocytoma was made.

Following excision of the intracranial tumor, our patient underwent adjuvant gamma knife radiosurgery with 24 Gy in two fractions to the tumor bed in the epidural space of the middle cranial fossa. No chemotherapy was administered. His postoperative course was uneventful with progressive resolution of the neurologic deficits. At follow-up 6.5 years later, he remains well with no signs of recurrence.

## Discussion

Our case highlights two notable aspects of IMM. First, it illustrates the importance of close collaboration between medical and dental professionals in such cases, in that it was not until a neurosurgery referral was made that a definitive diagnosis of IMM was made. Second, we provide detailed information regarding the appearance of this kind of tumor on MRI.

Including the case described here, 14 cases of IMM with detailed MRI descriptions are documented in the English literature (Table 1). However, our case of IMM is the first in which the initial presentation was to a dentist, which highlights the fact that a mass lesion in Meckel’s cave can mimic temporomandibular disorder by manifesting clinically as pain and malocclusion, leading the patient to visit a dentist first. Observed from one side only, the lesion was difficult to identify. Subsequent examination prompted further investigation to find the underlying cause. The final diagnosis of IMM was reached and satisfactorily managed only by collaboration between dental and medical practitioners.

**Table 1:** Reported cases of intracranial meningeal melanocytoma

First author and Reference number	Age/Sex	Location	Symptoms	Pattern of MRI signal intensity
Hamasaki [2]	59/M	Left cerebellopontine angle	Dizziness, headache, vomiting	High on T1, low on T2
Offiah [3]	25/F	Cisterna magna and posterior part of the foramen magnum	Headache, nausea, vomiting	High on T1, low on T2
Chen [4]	41/F	Right Meckel’s cave	Numbness of the right side of the face	High on T1, low on T2
Faro [11]	30/F	Adjacent to the left cavernous sinus and lesser wing of the left sphenoid bone	Severe frontal headache	Slightly increased signal in relation to adjacent white matter on T1, and low signal similar to adjacent cortical bone on T2
Srirama Jayamma [5]	62/M	Sulcal spaces	Episodic falls, difficulty in walking, brief loss of consciousness	High on T1 and low on T2
Lee [6]	45/M	Within the dura at the level of C1	Increasing pain around the neck	High on T1 and low on T2
Lee [7]	15/F	Left middle cranial fossa	Left facial hyperesthesia and paresthesia, diplopia	High on T1 and low on T2
Lin [8]	27/M	Frontal lobe	Headache and diplopia	High on T1 and low on T2
Painter [9]	35/M	Throughout the spinal canal, most prominent from C4 to T1	Headaches	High on T1 and low on T2
Pan [1]	36/M	Right cavernous sinus and the gyrus rectus	Headache accompanied by right eyelid ptosis	High on T1 and low on T2
Ruelle [10]	62/M	C5–C7	Slight weakness of the lower extremities and paresthesia on both hands	High on T1 and low on T2
de Tella Jr [12]	35/M	Around the optic nerve	Proptosis of the right eye	Isointense on T1, no change in intensity on T2
Tregnago [13]	28/M	In the inferior and lateral aspects of the right orbit	Proptosis of the right eye	Isointense to hyperintense on T1, predominantly isointense on T2
This study	39/M	Meckel’s cave	Numbness on left side of the face, pain, and malocclusion	High on T1, low on T2

*F* female, *M* male, *MRI* magnetic resonance imaging, *T1* T1-weighted images, *T2* T2-weighted images

The most prominent radiologic feature in this case was the pattern seen on MRI, namely, high signal intensity on T1-weighted images and low signal intensity on T2-weighted images compared with gray matter, which suggests melanoma or melanocytoma arising in the intracranial region. Eleven of the 14 IMM cases summarized in Table 1 showed the same MRI pattern of high signal on T1-weighted images and low signal on T2-weighted images [1–10], and the others demonstrated a similar signal pattern [11–13]. Knowledge of the characteristic imaging features of this infrequently encountered tumor, particularly the signal characteristics on MRI, can greatly assist in narrowing the differential diagnosis. The differential diagnosis is reported to include pigmented meningioma, melanotic schwannoma, and primary or secondary malignant melanoma [2]. Like meningioma, melanocytoma tends to be a solitary lesion, is often attached to the underlying dura, and may be locally invasive. IMM tends to occur in the posterior fossa and in the cerebellopontine angle, so may be difficult to differentiate from schwannoma [2]. Consistent with the reports by Hamasaki *et al.* [2] and Offiah and Laitt [3], our case showed homogeneous hyperdensity compared with gray matter on CT images.

## Conclusions

The case presented here underscores two important clinical issues in the diagnosis and treatment of IMM, namely, the importance of collaboration between medical and dental practitioners and the distinctive pattern of signal intensities on MRI. An interdisciplinary approach should be considered when such cases are encountered.

## Abbreviations

CT: Computed tomography
IMM: Intracranial meningeal melanocytoma
MRI: Magnetic resonance imaging
